# Transplacental Exposure to AZT Induces Adverse Neurochemical and Behavioral Effects in a Mouse Model: Protection by L-Acetylcarnitine

**DOI:** 10.1371/journal.pone.0055753

**Published:** 2013-02-07

**Authors:** Anna Rita Zuena, Chiara Giuli, Aldina Venerosi Pesciolini, Antonella Tramutola, Maria Antonietta Ajmone-Cat, Carlo Cinque, Giovanni Sebastiano Alemà, Angela Giovine, Gianfranco Peluso, Luisa Minghetti, Raffaella Nicolai, Gemma Calamandrei, Paola Casolini

**Affiliations:** 1 Department of Physiology and Pharmacology “Vittorio Erspamer”, I Faculty of Medicine, Sapienza University of Rome, Rome, Italy; 2 Section of Neurotoxicology & Neuroendocrinology, Department of Cell Biology and Neurosciences, Istituto Superiore di Sanità, Rome, Italy; 3 Section of Experimental Neurology, Department of Cell Biology and Neurosciences, Istituto Superiore di Sanità, Rome, Italy; 4 Therapeutic Area Life Cycle Management, Corporate R&D Sigma-Tau S.p.A., Pomezia, Rome, Italy; 5 Institute of Biochemistry of Proteins, CNR, Naples, Italy; Western University of Health Sciences, United States of America

## Abstract

Maternal-fetal HIV-1 transmission can be prevented by administration of AZT, alone or in combination with other antiretroviral drugs to pregnant HIV-1-infected women and their newborns. In spite of the benefits deriving from this life-saving prophylactic therapy, there is still considerable uncertainty on the potential long-term adverse effects of antiretroviral drugs on exposed children. Clinical and experimental studies have consistently shown the occurrence of mitochondrial dysfunction and increased oxidative stress following prenatal treatment with antiretroviral drugs, and clinical evidence suggests that the developing brain is one of the targets of the toxic action of these compounds possibly resulting in behavioral problems. We intended to verify the effects on brain and behavior of mice exposed during gestation to AZT, the backbone of antiretroviral therapy during human pregnancy. We hypothesized that glutamate, a neurotransmitter involved in excitotoxicity and behavioral plasticity, could be one of the major actors in AZT-induced neurochemical and behavioral alterations. We also assessed the antioxidant and neuroprotective effect of L-acetylcarnitine, a compound that improves mitochondrial function and is successfully used to treat antiretroviral-induced polyneuropathy in HIV-1 patients. We found that transplacental exposure to AZT given per os to pregnant mice from day 10 of pregnancy to delivery impaired in the adult offspring spatial learning and memory, enhanced corticosterone release in response to acute stress, increased brain oxidative stress also at birth and markedly reduced expression of mGluR1 and mGluR5 subtypes and GluR1 subunit of AMPA receptors in the hippocampus. Notably, administration during the entire pregnancy of L-acetylcarnitine was effective in preventing/ameliorating the neurochemical, neuroendocrine and behavioral adverse effects induced by AZT in the offspring. The present preclinical findings provide a mechanistic hypothesis for the neurobehavioral effects of AZT and strongly suggest that preventive administration of L-acetylcarnitine might be effective in reducing the neurological side-effects of antiretroviral therapy in fetus/newborn.

## Introduction

Since 1994, effective reduction in maternal-fetal HIV-1 transmission has been achieved by administration of antiretroviral (ARV) agents to HIV-1-infected women during pregnancy and to their newborns during early neonatal period [1]. The benefits deriving from the introduction of prophylactic ARV therapy are unquestionable but there is still considerable uncertainty on the potential long-term adverse effects of ARV agents on exposed children, in particular as for nucleoside reverse transcriptase inhibitors (NRTIs) [2]. In US and Europe, standard protocols of care for pregnant seropositive women and their newborns always include the NRTI zidovudine (AZT), alone or in combination with another NRTI, a non-NRTI or a protease inhibitor [3], [4].

Toxic effects of chronic treatment with AZT-based therapies have been widely documented in HIV-1 positive adults, including liver failure, lactic acidosis, myopathy, and neuropathy. These adverse effects have been mainly related to the ability of NRTIs, secondary to their antiviral action [5], to interfere with mitochondrial function in different target organs [6]–[8]. Specifically AZT induces alterations in mitochondrial structure and function by (a) direct inhibition of mtDNA replication and repair via inhibition of mtDNA polymerase *γ*; (b) alterations in cellular metabolism affecting oxidative phosphorylation enzyme activity and generation of reactive oxygen species; (c) mutations via incorporation of the NRTI into mtDNA and replication blockage [9], [10].

Results from clinical and cohort studies in offspring of women exposed to NRTIs during pregnancy, though excluding major adverse effects [4], have clearly shown the occurrence of subclinical mitochondrial dysfunction [11], [12], whose long-term repercussion on high-requiring energy tissues, such as the heart and the brain, is still a matter of concern. In a study carried out in a small number of infants born to HIV-1-positive women, quantification of mtDNA in cord blood evidenced mitochondrial damage and mtDNA depletion in NRTI-exposed infants when compared to unexposed infants [13]. A prospective study carried out in a large cohort of non-infected children within the French Pediatric Cohort reported the occurrence of symptoms compatible with mitochondrial dysfunction in a significant proportion of NRTI-exposed infants [14]. Such symptoms were primarily neurologic and included cognitive delay, motor disturbances, white-matter alteration in Magnetic Resonance Imaging and increased risk to develop febrile seizures, associated to deficits in one of the mitochondrial respiratory chain complexes [14]–[16]. Since AZT is central to highly active ARV therapy for reducing mother-to-child transmission of HIV-1 [4], [17], it is important to determine the nature and magnitude of the long-term effects of *in utero* AZT exposure as well as the mechanisms underlying the toxicities of this compound. However, in epidemiological studies the methodological issues related the potential effects of antiretroviral medications on neurobehavioral development are very complex, given the numerous co-morbidity factors that may influence neurobehavioral outcome in this specific group of children. These factors include, among the others, *in utero* exposure to maternal pro-inflammatory cytokines released during HIV infection, prenatal exposure to drug of abuse, family disruption, mental health problems in parents and birth complications [18]. Animal models represent a useful tool to overcome the methodological and ethical constraints implicated in human studies, and to investigate the etiology of antiretroviral drugs' toxicities in the absence of other confounding factors. A number of animal studies have been devoted to address this issue. These studies have clearly shown that developmental exposure to AZT produces both early and delayed behavioral changes in offspring. The behavioral endpoints affected by transplacental exposure to AZT include sensor and motor maturation, learning abilities, social/aggressive behavior, exploration levels [19]–[30]. Knowledge of the neural bases of behavioral AZT toxicity is so far very limited, but it has been clearly shown that at doses comparable to those used in clinical practice, AZT acts as a mitochondrial toxin in rodents and non-human primates [31]–[36]. Extensive evidence links dysfunctions of mitochondrial energy supply and the resulting oxidative stress to excessive release of glutamate [37] the major excitatory neurotransmitter in the mammalian central nervous system (CNS). By acting on ionotropic (iGlu) and metabotropic (mGlu) receptors, glutamate has a wide array of effects, ranging from modulation of learning and memory capacities [38]–[40], neuroendocrine secretion of glucocorticoids [41] and regulation of synaptic transmission and plasticity in the CNS [42], [43]. On these bases, we hypothesize that the behavioral alterations found in rodents exposed to AZT in utero are linked to oxidative stress resulting from AZT mitochondrial toxicity through the involvement of the glutamate system.

In order to assess this hypothesis we first evaluated in adult mice exposed *in utero* to AZT at clinical relevant doses spatial learning capacities, corticosterone response to acute stress, hippocampal glutamate receptors expression and oxidative stress, the latter by means of isoprostane and oxidized proteins level measurement. Furthermore, considering the implication of early oxidative stress induced by AZT in development of delayed neurobehavioral alterations, we evaluated whether the administration during pregnancy of a neuroprotective agent such as L-acetylcarnitine (LAC), capable of protecting mitochondria from damage induced by different noxa, was able to protect the transplacental AZT-exposed offspring from the CNS toxicity of this drug and, consequently, from the resulting behavioral impairment.

LAC, the acetyl ester of L-carnitine is a naturally occurring endogenous compound in all mammalian species present in relatively high levels in the brain, in particular in hypothalamus and hippocampus. When systemically administered, LAC readily crosses the blood-brain barrier influencing brain metabolism. LAC neuroprotective effects rely on improved mitochondrial energetic function, potentiation of antioxidant activities, stabilization of membranes, modulation of protein and gene expression [44].

Clinical studies have shown that treatment with LAC improved NRTI-induced polyneuropathy symptoms including pain, paraesthesia and numbness in HIV-1-positive patients [45]–[48]. However, the potential efficacy of LAC in protecting from the side effects of the prophylactic ARV therapy in pregnancy has never been studied so far.

## Materials and Methods

### Ethics Statement

This study was carried out in accordance with the Italian Animal Welfare legislation (art 4 and 5 of D.L. 116/92) that implemented the European Committee Council 106 Directive (86/609/EEC). The Italian Ministry of Health specifically approved the protocol of this study on 12/21/2009, Authorization n° 224/2009-b to G.C.

### Animals and housing conditions

Adult male (n = 30) and virgin female (n = 60) mice of the out bred Swiss-derived strain (CD-1) were purchased from Charles River. Upon arrival at the laboratory, the animals were housed in an air-conditioned room (temperature 21±1°C, relative humidity 55±5%) with a reversed 12∶12 light cycle (light on at 19.00 h, light off at 07.00 h). Water and food were available *ad libitum*. Females were group-housed (4 per cage) for 7 days to coordinate their estral cycle. After that, pairs of female mice were housed with a single sexually experienced male mouse. Females were daily inspected for the presence of a vaginal plug (gestational day 0; GD0) and then individually housed in Plexiglas cages (33×13×14 cm). Pregnancy rate was about 75%. The litters were culled at birth to four females and four males, to maintain adequate litter composition. Only male offsprings were used in this study.

### Experimental procedures

**Figure 1:**
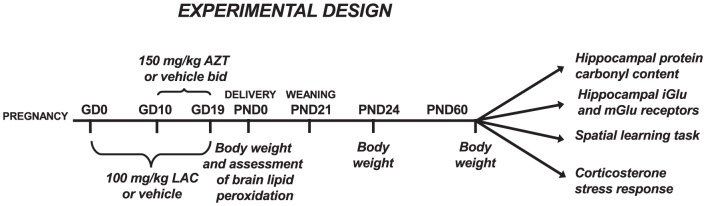
Experimental design of the study.

The experimental design of this study is depicted in Fig. 1. AZT was purchased from Sigma-Aldrich and LAC was provided by Sigma-Tau S.p.A. AZT was dissolved in bidistilled water and LAC in saline.

Following finding of the vaginal plugs 45 females were randomly assigned to one of the two subcutaneous (s.c.) treatments with either LAC (100 mg/kg, in a volume of 1.25 ml/kg) or vehicle (saline, same volume as LAC) that were administered daily from GD0 to the day of delivery. Starting on GD10, pregnant mice in each vehicle and LAC group were also administered by oral gavage (p.os) with either water (3.3 ml/kg) or AZT (150 mg/kg, same volume) in the morning and evening till the day of delivery. The resulting treatment groups were as follows: Control (saline s.c.+water p.os), LAC (LAC s.c.+water p.os), AZT (saline s.c.+AZT p.os) and AZT+LAC (AZT p.os.+LAC s.c.)

The AZT dose administered has been selected after multi-dose studies and taking into account the results of other groups' and ours studies in rodents, which identified doses producing behavioral effects and/or mitochondrial dysfunction in absence of significant reproductive or morphological effects in the mother and offspring [22], [29].

In order to measure the AZT levels in blood and brain, blood was withdrawn from the orbital plexus after delivery in five mothers of AZT group at the end of pregnancy, in a period between 8 and 12 hours after the last AZT treatment. Four pups from each of the same litters were sacrificed at birth, and their plasma and brain hemispheres collected and pooled (n = 5).

Females' body weight was monitored daily during pregnancy. Proportion of term pregnancies, gestation length, litter size, sex ratio and neonatal mortality were also measured to exclude potential effects of the treatment on reproductive performances. The day of birth was defined as postnatal day 0 (PND0). At this age, before litter culling, eight pups from each treatment group were sacrificed for F2-isoprostane measurement in the total brain homogenate. Weaning took place at 21 days of age (PND21) and male offspring of each group housed in two per cage. They were maintained under controlled environmental conditions until 2 months of age (PND60) when they were assigned to water maze spatial learning test, plasma corticosterone assay, protein oxidation and glutamate receptor analysis. Body weights were recorded at birth (PND0), after weaning (PND24) and before performance of the spatial learning task (PND60).

### Determination of AZT Concentrations in Plasma and Brain Homogenate

Plasma and brain homogenate samples were analyzed separately using spiked standards in blank plasma and brain homogenate. Commercial available AZT (Sigma-Aldrich) was used as internal standard. For plasma, 100 µl samples were spiked with 100 ng of internal standard. The samples were subjected to liquid-liquid extraction using 8 parts of ethyl acetate for 1 part of sample and vortexed vigorously for 5 min. The mixture was centrifuged at 14,000 rpm at room temperature for 10 min. The supernatant was transferred to clean glass tubes, dried under a flow of liquid nitrogen, and reconstituted in 100 µl of mobile phase, and 30 µl was injected onto the HPLC column. Whole brain was homogenized in 3 volumes of 5% bovine serum albumin in PBS using a Dounce homogenizer (7 ml; Kontes Glass); 500 µl of brain homogenate was spiked with 50 ng of internal standard, and sample preparation was similar to that of plasma. The mobile phase for AZT analysis consisted of buffer (50 mM ammonium phosphate, 50 mM sodium citrate buffer, and 10 ppm sodium azide, pH 6.5) and methanol (82∶18) at a flow rate of 0.2 ml/min, and UV absorbance was measured at 266 nm. The plasma and brain concentrations are reported as nanograms per milliliter and nanograms per gram of brain tissue, respectively.

### HPLC Analysis

AZT determination was performed by HPLC analysis using a Hypersil-BDS column (C-18, 2.1 mm×150 mm, 5 µM; Thermo Electron Corporation) maintained at 40°C using a Shimadzu column oven (CTO-10Avp). The HPLC system consisted of a Shimadzu pump (LC-10ATvp), flow control valve (FCV-10ALvp), degasser (DGU-20A5), auto injector (SIL-10ADvp), system controller (SCL-10Avp), and detector (SPD-10Avp).

### F2-isoprostane measurement

On PND0 the levels of the F_2_-isoprostane (F_2_-IsoP) were measured in brain homogenates, as previously described [49]. Briefly, brains were weighed and homogenized in 50 mM Tris buffer, pH 7.5 (1 mg/0.1 ml), containing the anti-oxidant 10 µM BHT to block spontaneous oxidation. Homogenates were vigorously vortexed and incubated for 5 min on ice before centrifuging at 14,000 rpm for 45 min at 4°C. Supernatants were collected and stored at −80°C until required. 15-F_2t_-IsoP, the major member of F_2_-IsoP family, was measured by a specific enzyme immunoassay (Cayman Chemical), according to the manufacturer's instructions. Detection limit was 2 pg/ml; anti-15-F_2t_-IsoP antibody cross-reactivity with other iso-prostaglandins was less than 0.15%.

### Detection of Oxidized Proteins

Protein oxidation was measured in the same homogenates utilized for western blot of glutamate receptors in 2-month-old mice. Oxidized protein was detected by using an oxidized protein detection kit (OxyBlot, Chemicon International). The OxyBlot provides reagents for sensitive immunodetection of carbonyl groups, which is a hallmark of the oxidation status of proteins. The procedure was performed according to manufacturer's recommendation. Briefly, 30 µg of hippocampal homogenates were derivatized with or without 2,4-dinitrophenylhydrazine (DNPH) and samples loaded onto a 12% SDS-PAGE gel. After separation, proteins were electrotransferred to a nitrocellulose membrane and incubated with a primary antibody against the derivatized carbonyl groups followed by incubation with a horseradish peroxidase-conjugate goat anti-rabbit antibody. The oxidized proteins were visualized by using an enhanced chemiluminescence system (Amersham Biosciences). The relative optical densities were quantified using the NIH ImageJ medical imaging software. No immunoreactivity was detected in the non-DNPH derivatized brain homogenates.

### Water Maze procedure

On PND60 mice from each treatment group were tested in the Morris water maze for their spatial learning ability, applying a one-day protocol that has been successfully applied to different strains of mice [50]. The apparatus consisted of a circular pool (diameter 110 cm, height 60 cm) located in a test room with white walls with several cues on them. The pool, with its inner surface painted black, was filled to a depth of 40 cm with water (maintained at 25±1°C), covering an invisible (black) 10-cm square platform. The platform was located approximately 0.5 cm below the surface of the water. The pool was virtually divided into 4 quadrants (North, South, East, or West) and the platform placed at a fixed position in the center of the North quadrant. A single-day training procedure was carried out, and each subject underwent four four-trial sessions of training, with each session separated by 30 min. On each trial, the subject was gently released in the water with its head facing the pool wall from one of quadrants. The order of the starting quadrant was changed in each session and trial. A maximum of 60 sec was allowed, during which the mouse had to find the platform, climb onto it and allowed to remain there for 10 sec. If the animal did not find the platform, it was gently guided with a grid and allowed to stay for 10 sec.

A video camera above the center of the pool was connected to a computerized tracking system that recorded and analyzed animal behavior (San Diego Instruments). The time of escape onto the platform and the swimming speed were measured. After the last trial of the last session of training, animals were submitted to a single 60 sec “probe trial” in which the platform was removed from the pool. The animal started the probe trial in the south quadrant and the time that it swam through the north quadrant, where the platform had been previously located, provided a measure of learning accuracy in recalling the former position of the platform. A session with a visible platform was performed at the end of the learning task to assess the swimming speed of the different groups of animals.

### Corticosterone secretion after acute restraint stress

A second group of mice from each treatment group was used for assessment of plasma corticosterone levels in basal conditions and following 15 min of acute restraint stress. The procedure was performed in the animal facility at 9:00 AM. Restraint stress was performed as follows: the mouse was removed from its cage and placed in an adjustable Plexiglas restraint device for 15 min under a very bright light. Blood samples were collected from the tail tip in heparinized capillary tubes at the beginning (basal value) and at the end of the restraint procedure (stress value). At the end of the procedure the mice were immediately returned to their cages. Blood samples were centrifuged at 1900×g at 4°C for 20 min; plasma was removed and kept frozen at −20°C until assay. Plasma corticosterone concentrations were determined by radioimmunoassay (MP Biomedicals). The cross-reactivity of the polyclonal corticosterone-antisera with respective related substances was negligible. The inter- and intra-assay coefficients of variance were 7% and 4%, respectively, with a detection limit of 0.01 µg/100 ml.

### Western blot analysis

A third set of mice was sacrificed on PND60 to assess expression of iGlu and mGlu receptors. For the iGlu receptors we assessed AMPA Glu1 and Glu2 subunit, and NMDA NR1 subunit. For the mGlu receptors we measured both group I mGluR1 and mGluR5 subtypes and group II mGlu2/3 subtypes. Mice were killed by decapitation and brains rapidly removed; hippocampi were dissected and stored at −80°C. On the day of the experiment, tissue was homogenized at 4°C with a polytron in 500 µl of 100 mM Tris buffer, containing phenylmethylsulfonyl fluoride 1 mM, leupeptin 10 µg/ml and aprotinin 10 µg/ml pH 7.2. Protein concentrations were determined using the Bradford protein assay. Thirty micrograms of protein were re-suspended in sodium dodecyl sulfate (SDS)-bromophenol blue loading buffer with 0.5 M dithiothreitol. The samples were separated on 8% SDS-polyacrylamide gels (Amersham Bioscience) and after electrophoresis (Mini-PROTEAN 3 System, Bio-Rad), the proteins were transferred to nitrocellulose membranes (Amersham Bioscience) using a system of mini transblot cell (BioRad) overnight. After transfer, blots were incubated in a solution (blocking solution) containing Tris-buffered saline (TBS), 10% (w/v) Tween-20, 1% (w/v) non-fat milk and 1% (w/v) bovine serum albumin. Subsequently, blots were incubated overnight with rabbit anti-mGluR1a (1∶1000), anti-mGluR5 (1∶1000), anti-mGluR2/3 (1∶1000), anti-NR1 (1∶1000), mouse antiGlu1 (1∶500) (Upstate Biotechnology) and mouse antiGlu2 (1∶500; Chemicon International) in blocking solution at 4°C. After incubation with the primary antibody, the blots were incubated with horseradish peroxidase-conjugated goat anti-rabbit or anti-mouse antibodies (1∶5000; Amersham Bioscience) for 1 h at room temperature (21°C±2). To ensure that each lane was loaded with an equivalent amount of protein, the blots were probed with an anti-actin serum (1∶1000; Sigma) overnight at 4°C. Subsequently, blots were incubated with horseradish peroxidase-conjugated goat anti-mouse antibodies (1∶5000; Amersham Bioscience) for 1 h at room temperature. Immunoreactive bands were visualized with an enhanced chemiluminescence system (Amersham Biosciences). After immunoblotting, digitized images of bands immunoreactive for target (mGluR1, mGluR5, mGluR2/3, NR1 or Glu1) and control (actin) molecules were acquired and the area of immunoreactivity corresponding to each band was measured using the NIH ImageJ medical imaging software. A ratio of target to actin was then determined, and these values were compared for statistical significance.

### Statistical analysis

Body weight data were analyzed separately for each age point by one-way ANOVA. Latencies to reach the platform in the Morris water maze were analyzed by three-way ANOVA for repeated measures (treatment×trial×session with repeated measures on trials and sessions). Each litter in each final treatment group contributed with a single subject to spatial learning task, corticosterone assessment and measurement of glutamate receptors/protein carbonyl content. The ANOVA analyses were always followed by Fisher's LSD *post-hoc* comparisons. Probe trial, swimming speed, plasma corticosterone concentrations and immunoblotting data were analyzed by Student's t-test. The level of significance was set at p<0.05.

## Results

### AZT administered during gestation reaches the brain of fetuses

In female mice after delivery, mean AZT plasma concentration was 8.6±1.0 ng/ml, a value comparable to that found in women treated during pregnancy following clinical protocols [51]. Plasma concentration of AZT in pups was undetectable, but significant AZT levels were found in the brain (70.2±6.1 ng/mg of brain tissue), indicating the transplacental passage of AZT to the brain of fetuses (AZT vs Control, p<0.05 Fisher's LSD *post-hoc*).

### AZT induced decrease of body weight at birth is not prevented by LAC

Proportion of term pregnancies, gestation length, litter size, sex ratio and neonatal mortality was not affected by prenatal treatment with AZT or LAC. The mean body weight of the offspring at different ages is shown in Table 1. At birth (PND0) AZT treatment significantly decreased body weight (main treatment effect F_3,36_ = 11.58, p<0.05; AZT vs Control, p<0.05 Fisher's LSD *post-hoc*), an effect that was not prevented by administration of LAC in the AZT+LAC group (AZT+LAC vs Control, p<0.05 Fisher's LSD *post-hoc*). LAC *per se* did not modify pups' body weight at birth, but increased it on PND24 (LAC vs all other groups, p<0.05 Fisher's LSD *post-hoc*). At PND60, mean body weight was comparable in the four experimental groups.

**Table 1:** Effect of transplacental exposure to AZT and LAC on body weight recorded at different ages in male offspring.

	Control	AZT	LAC	AZT+LAC
PND0	1.83±0.04	1.63±0.03*	1.89±0.06	1.57±0.05*
PND24	15.01±0.81	14.49±0.68	17.04±0.77#	14.43±0.51
PND60	38.69±1.17	37.04±1.75	39.71±1.13	37.46±1.24

AZT treatment affected body weight at birth irrespectively of LAC administration, but this effect disappeared by PND 24. LAC treated mice was generally heavier than Control group. (Fisher's LSD *post-hoc* after one way ANOVA:

* p<0.05 vs Control;

# p<0.05 vs all other groups). Values are means ± S.E.M (n = 9–11 mice per group).

### AZT- induced impairment of spatial learning and memory is counteracted by LAC treatment

**Figure 2:**
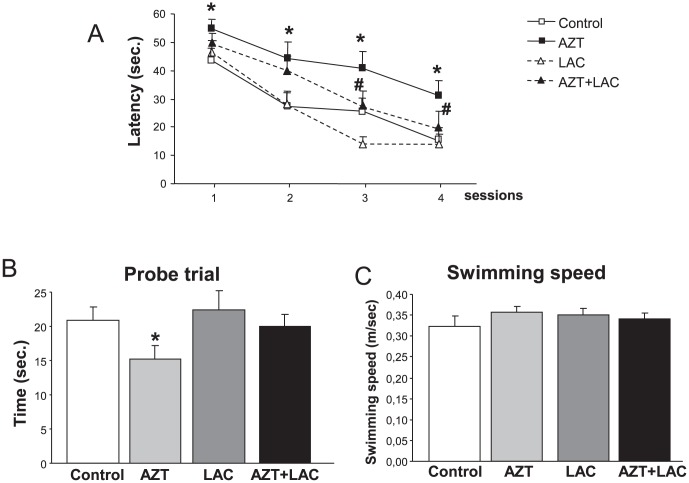
Effect of AZT and LAC treatments on spatial learning and memory assessed in the Morris Water Maze. **A) Training sessions:** Latencies to reach the hidden platform throughout the four sessions of the task. The higher latencies shown by AZT-treated mice in comparison to Control mice throughout the four sessions clearly indicate a learning impairment (* p<0.05, Fisher's LSD *post-hoc* performed on significant three-way ANOVA interaction), counteracted by LAC treatment, as the AZT+LAC group had latencies comparable to the Control group in the 3rd and 4th session, and significantly different from those of the AZT group (# p<0.05). LAC treatment alone did not modify learning abilities. **B) Probe trial:** AZT-treated mice spent less time than Control mice in the quadrant where the platform was located during training (* p<0.05 AZT vs Control after *t* test), indicating a deficit in memory recall. AZT+LAC mice did not differ from Controls. **C**) No treatment-induced difference was recorded in the swimming speed assessed in a trial with a visible platform. Values are expressed as means ± S.E.M (n = 7–8 mice per group).

Figure 2A shows the latencies to reach the hidden platform throughout the four sessions of the Morris water maze. ANOVA for repeated measures evidenced a significant main effect of the treatment received (F_3,26_ = 5.95, p<0.05) and a significant three way interaction treatment×session×trial (F_3,27_ = 1.89, p<0.05). *Post-hoc* analysis showed that subjects receiving prenatal AZT treatment displayed a significant increase in escape latency in comparison to the Control group in all the four sessions. The AZT+LAC group had escape latencies comparable to the Control group and significantly different from those of the AZT group in the 3^rd^ and 4^th^ session, suggesting that pre-treatment with LAC significantly improved the AZT-induced acquisition deficit. Finally, LAC treatment alone did not modify learning abilities.

During the probe trial (Fig. 2B) AZT subjects spent significantly lower time than Control mice in the quadrant where the hidden platform was located during the acquisition trials (t = −2.05, p<0.05), indicating that they were impaired in recalling the former position of the platform. Notably, performances of AZT+LAC mice in probe trial did not differ from those of Controls.

No treatment-induced difference was recorded in the swimming speed of the different groups of mice (Fig. 2C).

### LAC treatment protects from AZT-induced enhancement of stress response

**Figure 3:**
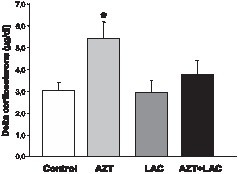
Effect of AZT and LAC treatments on plasma corticosterone secretion measured after 15-min restraint stress. In basal conditions, no significant differences were observed in plasma corticosterone levels between groups (see text for details), while following acute stress AZT-treated mice had markedly enhanced corticosterone release in comparison to Control mice (* p<0.05 AZT vs Control, Student's t test). LAC administration protected from AZT-induced increase of corticosterone. Corticosterone levels were calculated as delta between restraint stress values and basal values. Values are expressed as means ± S.E.M. (n = 7 mice per group).

In basal conditions, no significant differences were found in plasma corticosterone levels between groups (Controls = 4.10±0.41 µg/dl; AZT = 3.47±0.37 µg/dl; LAC = 3.45±0.65 µg/dl; AZT+LAC = 3.34±0.29 µg/dl). Student's *t*-test of plasma corticosterone stress response, calculated as differences (delta) between restraint stress values and basal values, revealed that mice in the AZT group had higher plasma corticosterone concentration in respect to the Control group (t = 2.76, p<0.05). Of note, the AZT+LAC animals showed levels of corticosterone similar to the control mice suggesting that the prenatal treatment with LAC was able to protect the development of hypothalamus pituitary adrenal (HPA) axis from the effects of AZT (Fig. 3).

### Reduced expression of hippocampal metabotropic and ionotropic Glu receptors caused by AZT is counteracted by LAC treatment

**Figure 4:**
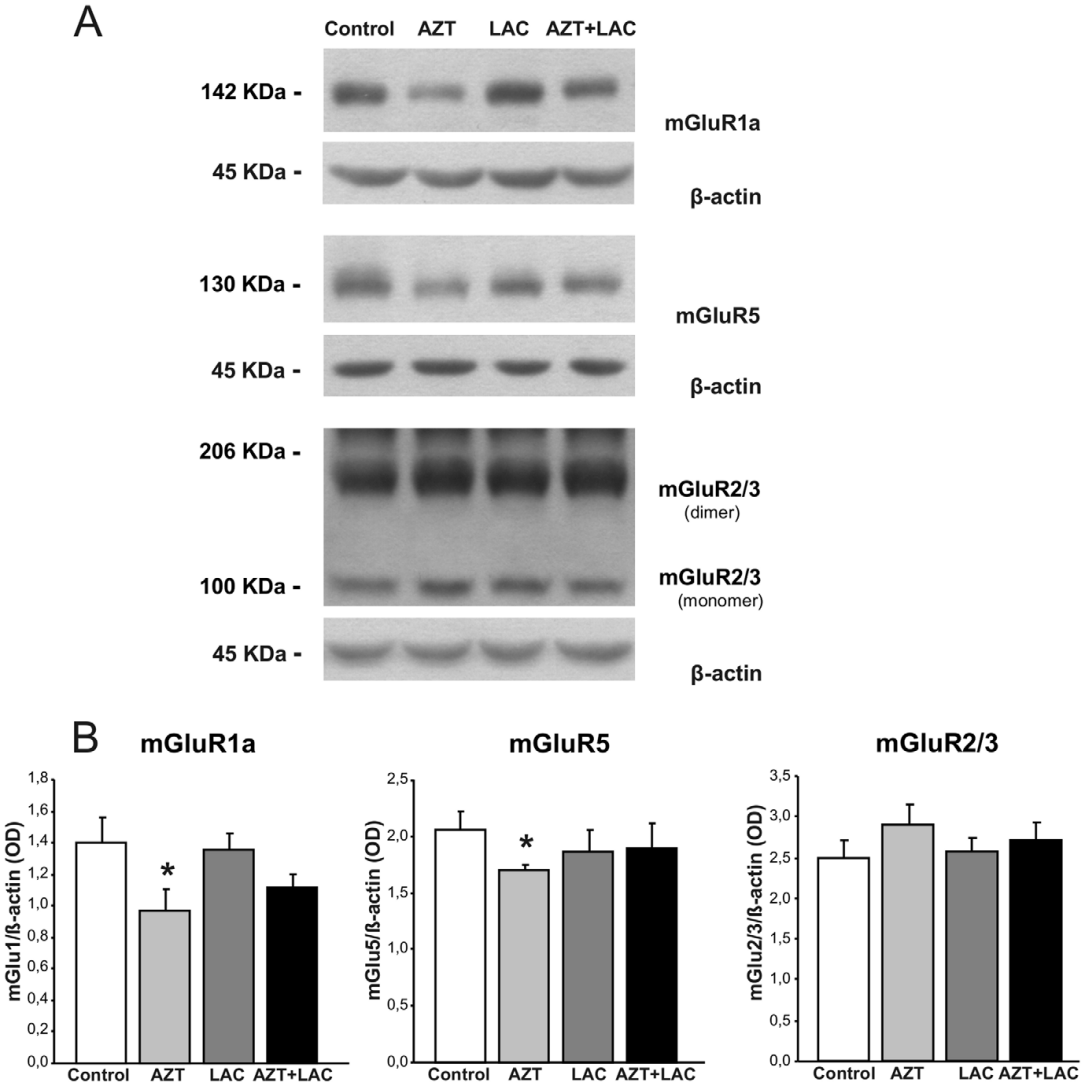
Effect of AZT and LAC treatments on hippocampal mGlu receptors expression. (**A**) Representatives of immunoblots with mGlu1a and mGlu5 receptor antibodies showed a 142 kDa and 130 kDa bands, corresponding to receptor monomers, respectively. Blots with antibodies recognize an epitope common to mGlu2 receptors monomer(s) (100 kDa) and mGlu3 receptors dimers (206 kDa). (**B**) Results are expressed as the ratio of the optical density (OD) of the mGluR1a, mGluR5 or mGlu2/3 band and the β-actin band. The AZT prenatal treatment caused a significant reduction of mGlu1a and mGlu5 receptors expression in respect to Control mice (* p<0.05, Student's t-test) counteracted by LAC treatment as AZT+LAC mice differ from Control. No differences between groups were found in the mGluR2/3 (summary of OD monomers and dimers). Values are expressed as means ± S.E.M. (n = 6 mice per group).

The AZT prenatal treatment caused a significant reduction of mGlu1a receptor expression in respect to Control mice (p<0.05, Student's t-test, t = −2.32; Fig. 4). Interestingly, the mGlu1a receptors in AZT+LAC group showed a trend to increase (although not statistical significant) in respect to AZT animals and their expression did not differ from that found in the Control group. Therefore, LAC treatment appeared to counteract the AZT-induced reduction of mGlu1a receptor expression. This phenomenon, although less evident, was also observed for mGlu5 receptor expression: the AZT group showed reduced receptor expression compared to Control group (p<0.05, Student's t-test, t = −3.17), while the AZT+LAC group showed a trend to increase. For what concerns the mGlu2/3 receptor no differences between the four experimental groups were found.

**Figure 5:**
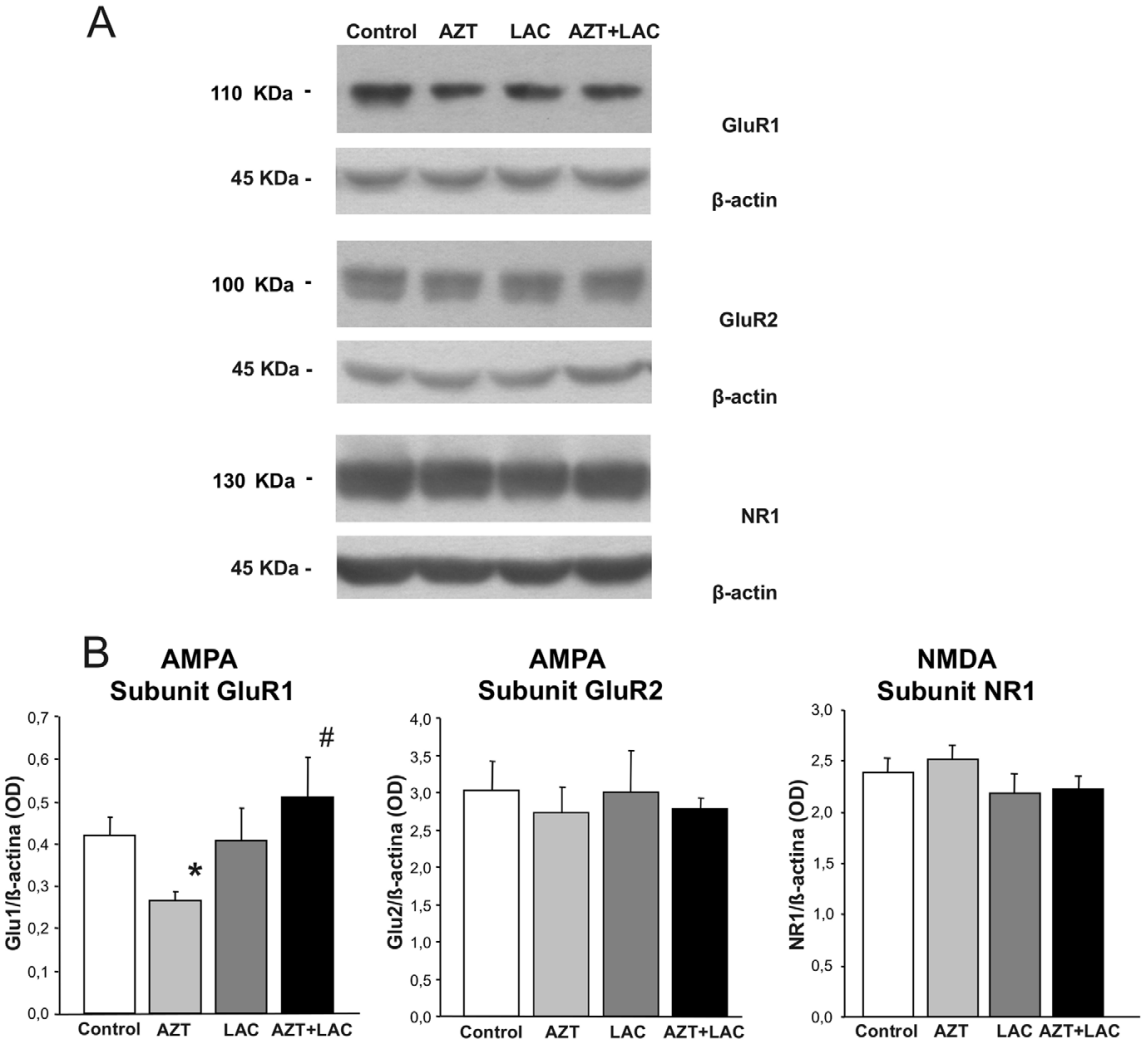
Effect of AZT and LAC treatments on hippocampal AMPA and NMDA receptors expression. (**A**) Representatives of immunoblots with GluR1 and GluR2 receptor antibodies showed a 110 kDa and 100 kDa bands corresponding to AMPA receptor subunits, respectively. Blot with NR1 receptor antibody showed a 130 kDa band, corresponding to NMDA receptor subunit. (**B**) Results are expressed as the ratio of the optical density (OD) of the GluR1, GluR2 and NR1 band and the β-actin band. AZT prenatal treatment caused a significant reduction of the GluR1 subunit of AMPA receptors expression in respect to Control mice (* p<0.05 AZT vs Control, Student's t-test) that was fully prevented by LAC treatment: in AZT+LAC mice the Glu1 subunit was increased in respect to AZT mice (# p<0.05 vs AZT, Student's t-test) and did not differ from Controls. Expression of the GluR2 subunit of AMPA and NR1 subunit of NMDA receptors was not affected by either treatment. Values are expressed as means ± S.E.M. (n = 6 mice per group).

In Fig. 5 protein expression relative to ionotropic receptors is shown. The analysis by Student's t-test confirmed that AZT prenatal treatment yielded a significant reduction of Glu1 subunit of AMPA receptor expression in respect to Control mice (p<0.05, Student's t-test, t = −3,43). Notably, the Glu1 subunit in AZT+LAC group showed a significant increase (p<0.05, Student's t-test, t = −2.59) in comparison with AZT mice and did not differ from Controls suggesting that LAC treatment prevented the reduction of receptor expression due to AZT. As for the AMPA GluR2 and NMDA NR1 subunits, no differences between groups were found.

### Increased levels of lipid peroxidation and oxidative stress caused by AZT are prevented by LAC treatment

**Figure 6:**
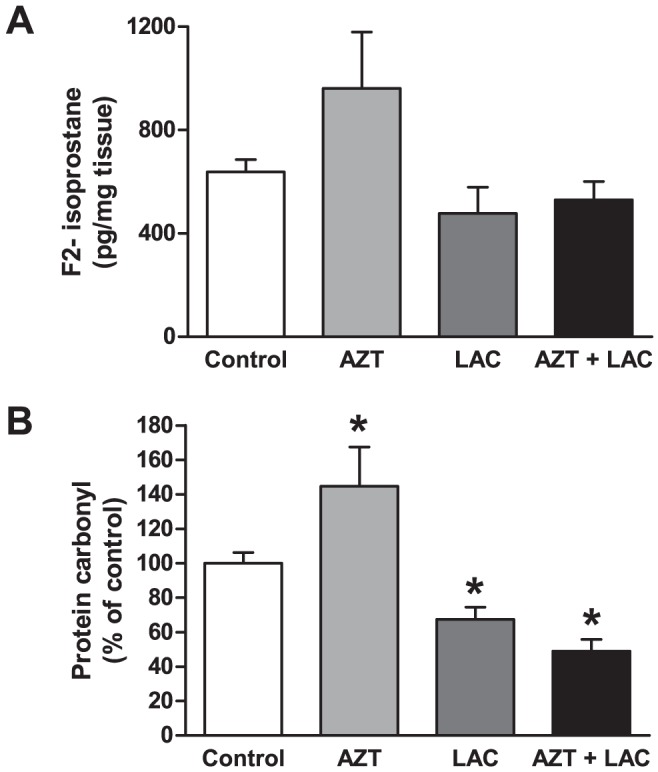
Effect of AZT and LAC treatments on brain oxidative stress in newborn and adult mice. (**A**) Levels of F2-Isoprostane, as marker of lipid peroxidation and oxidative stress, in brain homogenates from newborn mice. F2t-Isop levels in whole brain homogenates showed a trend to increase in samples from AZT prenatally-treated pups, but not in those from LAC or AZT+LAC groups. (**B**) Protein carbonyl content (Oxyblot) of hippocampal homogenates. The AZT prenatal treatment caused a significant increase of the protein carbonyl content in respect to Control mice. Both LAC and AZT+LAC groups showed a significant decrease of carbonyls in respect to Control mice indicating an antioxidant effect of the LAC treatment. (* p<0.05 vs Control, Student's t-test). Values are expressed as means ± S.E.M. (n = 6–8 mice per group).

Occurrence of free radical generation and oxidative stress was monitored at birth and at PND60. On PND0, we tested the levels of 15-F_2t_-IsoP, a reliable and sensitive marker of oxidative stress [52], suitable for oxidative stress evaluation in small size samples. Consistent with the oxidative stress hypothesis of AZT toxicity, 15-F_2t_-IsoP levels in whole brain homogenates (Fig. 6A) showed a trend to increase in samples from AZT prenatally-treated pups, but not in those from LAC or LAC+AZT groups. Protein carbonyl levels, which are considered a measure of protein oxidation and an index of oxidative stress [53]–[55], were measured by OxyBlot analysis of homogenates prepared from hippocampi of mice sacrificed at PND60, in parallel with glutamate receptor expression. In agreement with the tendency observed at PND0, a significant increase in carbonyls was found in the hippocampal homogenates from AZT prenatally-treated mice (45% of Control, p<0.05, Student's t-test, t = 2,15; Fig. 6). Significant decreases of protein carbonyls were detected in the hippocampus of mice treated with LAC and with the combination of AZT+LAC, as expected due to the antioxidant effect of LAC (p<0.05, Student's t-test, t = −3,45 for LAC, t = −5,07 for AZT+LAC; Fig. 6).

## Discussion

In the past years, reports of clinical signs suggestive of mitochondrial dysfunction in non-infected children exposed to NRTIs have prompted studies addressing the issue of developmental toxicity of such drugs and, in particular, of AZT.

Occurrence of subclinical mitochondrial dysfunction has been consistently shown by either clinical and experimental studies in different tissues and organs, including the brain [11]–[14], [36], [56]. In particular, a recent study provided evidence that *in utero* exposure to the NRTI, AZT and Lamivudine, is associated with brain mitochondrial impairment, which progresses over time and might possibly end up in delayed neurobehavioral effects [32]. In laboratory rodents, developmental exposure to AZT produces both early and delayed behavioral changes in offspring including alteration in sensorimotor maturation, social/aggressive behavior, responses to environmental stressors and learning abilities [19]–[30]. Altogether, these data pointed to the potential risk of sub-clinical side effects of ARV therapy on the developing brain that may go undetected in clinical and epidemiological studies. Thus, current recommendations call for long-term clinical follow-up for any child with *in utero* exposure to ARV drugs [17] to monitor significant side effect of ARV drugs that may augment risk in children and adolescents born to HIV-1-positive women, who represent a vulnerable group as for psychiatric health [18], [57]–[59].

The main results of the present preclinical study contribute to clarify this controversial issue, as they show that 1) transplacental exposure to AZT enhances oxidative stress and causes a consistent and permanent alteration of hippocampal glutammatergic neurotransmission functionally associated to a deficit in spatial learning and memory, 2) LAC administration from the beginning of pregnancy prevents/ameliorates the adverse effects of AZT on both neurochemical and behavioral parameters.

In line with previous findings in rodent models, we show that transplacental AZT exposure has significant long-term effects on behavior of exposed offspring. In particular, AZT treatment has detrimental effects on performances in the water maze task, one of the most widely used tests to measure hippocampal-dependent spatial-based learning and memory in rodent models with high translational value with respect to humans [60]. We observed a marked deficit in acquisition and memory recall, as both acquisition of the platform location and retrieval of the acquired information in the probe trial were impaired in AZT-treated mice. Since swimming speed was not affected by AZT treatment, we can exclude motor impairment possibly associated with the well-known effects of this drug on muscle tissue [61].

Different neurotransmitter systems have been implicated in the effects exerted by AZT and other ARV drugs on behavior but a correlation between specific neural mechanisms and the behavioral changes observed has not been attempted yet. Extensive evidence indicates that learning and memory components of spatial navigation in rodents rely on hippocampal synaptic plasticity [62]–[64] that is largely modulated by hippocampal mGlu [65], [66] and by AMPA receptors [67]. Our findings indicate that hippocampal glutamate neurotransmission is permanently affected by early AZT exposure and support a role of this important neurochemical pathway in the behavioral effects here reported.

The reduced expression of metabotropic group I and ionotropic Glu1 subunit of AMPA receptors in the hippocampus is well in agreement with the specific impairment of spatial learning and memory performances observed in AZT treated mice. Recent data strongly support a role for the different glutamate receptor subtypes in spatial cognition: administration to rats of mGluR1 antagonists affects synaptic plasticity and impairs acquisition in the Morris water maze [68], while blockade of mGlu5 receptors interferes with long-term potentiation and has detrimental effects on spatial learning [69]. As well as for metabotropic group I receptors, there is ample evidence that the dynamic regulation of AMPA receptors - which mediate most of the fast excitatory synaptic transmission - can change synaptic function and regulate storage of information [70], [71]. Based on our behavioral and neurochemical findings, we hypothesize that the lower expression of different subgroups of glutamate receptors in AZT mice results in reduced plasticity of hippocampal glutamatergic synapses and, as an ultimate consequence, in diminished capacity to encode relevant information during the spatial learning task.

Such effect could be further amplified by the enhanced corticosterone responsiveness to stress in AZT exposed mice. Our findings show that administration of AZT during pregnancy caused increased release of plasma corticosterone following acute restraint stress at adulthood. As water maze training induces release of high corticosterone levels shortly after completion of the task in mice [72], an abnormal physiological reaction to stress could interfere with both acquisition and consolidation of information in AZT mice. An inverse relationship between spatial learning and memory and elevated corticosterone levels has been widely demonstrated in rodents: exposure to mild stressor or moderate glucocorticoid levels facilitates spatial memory [73]–[76] while chronic stress or higher glucocorticoid levels exert detrimental effects on neuronal plasticity and spatial memory [77]–[79]. The increased corticosterone release after acute stress observed in AZT treated mice deserves further consideration, as it is suggestive of altered HPA axis response caused by early AZT exposure. A recent study indicates that chronic AZT is able to increase corticosterone release in adult rats possibly by a central action [80]. In a developmental perspective, AZT treatment could interfere with the physiological setting of HPA axis activity, thus reducing the capability to respond adaptively to environmental challenges. Previous studies from our laboratories indicate that mice exposed to AZT during development present abnormal reaction to novelty, and that pre- and/or postnatally AZT exposed adult male mice show maladaptive intraspecific social/aggressive behavior depending on the length of exposure [23], [24].

Overall, our findings confirm the behavioral effects of transplacental AZT in rodent models. More importantly, they point for the first time to a specific and permanent action of this drug on glutamate neurotransmission. We propose that such effect is linked to the well-known mitochondrial toxicity of AZT [11], [14], [31], [32], [34], [81]–[83]. NRTI-induced alterations in mitochondrial structure and function result from interference with various mechanisms involved in the normal maintenance of mitochondrial function. In particular, it has been suggested that AZT directly impairs the electron transport chain (ETC) thus increasing production of reactive oxygen species (ROS) and oxidative stress, which will eventually lead to a loss of the mtDNA integrity [84]. Our data confirm that transplacental exposure to AZT induces oxidative stress in the fetal brain and determines long-term effects on the oxidative status of brain tissue as two different oxidative stress markers, 15-F_2t_-isoP and protein carbonyl levels at birth and at adulthood, respectively, were enhanced in AZT-treated offspring.

F_2_- Isoprostanes are lipid peroxidation products; they are among the most sensitive in vivo biomarkers of oxidative stress and are the marker of choice for the evaluation of oxidative damage in small size samples such as newborn mouse brain [52]. Similarly, immunodetection of carbonyl groups provides a measure of oxidation status of proteins, and in turn, of oxidant injury. The increased levels of 15-F_2t_-isoP and protein oxidation are indicative of increased levels of ROS, likely deriving from AZT-dependent mitochondrial impairment, as described in several previous studies [5]–[8].

The evidence of early oxidative stress in AZT exposed offspring allows us to advance a mechanistic explanation for the endocrine and neurobehavioral effects here reported. Impairment of mitochondrial function and production of increased levels of ROS may reduce the capacity of the cell to generate ATP, and induce excess glutamate release at the synapse and, eventually, excitotoxicity [37]. In turn, an excess of glutamate release in the early stage of life results in overstimulation of glutamatergic receptors that can be possibly permanently modified in their expression and functionality. Besides, it should be considered that a significant portion of the control of HPA axis activity is mediated by glutamate [85]–[88] thus enhanced glutamate release during early development could result in lifelong alteration of HPA axis responsiveness. The link between AZT-induced oxidative stress in the fetal stage and the long-term consequences on neurobehavioral functions at the adult stage is strongly supported by the protective effect exerted by LAC. Indeed, we show that most of the alterations induced by AZT are prevented or reduced by administration of LAC from the beginning of pregnancy.

Several lines of evidence indicate that administration of antioxidant agents, including vitamin C, E and coenzyme Q10 prevents or alleviates oxidative stress and symptoms of AZT-induced myopathy in both rats and humans (see 61 for a review).

More relevant to our study, LAC is known to decrease oxidative stress by 1) exerting a protective effect on mitochondrial structure and function, making the ETC less prone for electron leak and superoxide production, and promoting mitochondrial biogenesis, as indicated by increased mitochondrial size and number [89]; 2) stimulating the endogenous cellular antioxidant defense mechanisms. These effects possibly rely on the ability of LAC to act as a donor of acetyl groups. In a recent work, a proteomic survey of protein acetylation identified that 20% of mitochondrial proteins presented with acetylation sites and these proteins included those involved in the tricarboxylic acid cycle, fatty acid β-oxidation, amino acid and carbohydrate metabolism, membrane transport, and ETC [90]. These data clearly show that acetylation can control the activity of mitochondrial enzymes and, since acetyl-CoA is the acetylation donor for all known acetyltransferases, the concentration of mitochondrial acetyl-CoA could be a limiting factor in the acetylation reaction. By increasing the acetyl-CoA content, supplemented LAC should increase the acetylation status of mitochondrial proteins thus improving mitochondrial function and integrity. Interestingly, low levels of circulating LAC have been found in HIV-1 patients experiencing a clinically manifest peripheral neuropathy while staying on AZT and other nucleoside analogue treatment [91], and in patients with AZT-induced mtDNA depletion [92]. Hence, LAC deficiency could play a role in neurotoxic effects of these drugs. AZT treatment reduces carnitine levels in blood and tissues [5], [6] and LAC treatment could mediate an improvement of mitochondrial dysfunction in a straightforward manner, namely, by replenishing carnitine levels.

Other clinical studies have shown that treatment with LAC improved NRTI-induced polyneuropathy symptoms in HIV-1-positive patients, including electrophysiological variables relating to motor conduction velocity [93]. In a morphological study in HIV-1 patients with distal symmetrical polyneuropathy, regeneration of all fiber types and in particular of small sensory fibers in the dermis, epidermis and sweat glands was observed after oral LAC treatment [94]. All this considered, LAC can be viewed as a pleiotropic molecule metabolically active, with neuromodulatory, neurotrophic, cytoprotective, and antioxidant activity in brain.

The use of ARV drugs to prevent mother-to-child transmission of HIV-1 infection is one of the most successful achievements in HIV-1 prevention and, based on several clinical and epidemiological studies, benefits of this therapy appear to outweigh its adverse side effects in exposed infant/children [2], [95]. However, there is still considerable uncertainty on the prevalence and severity of mitochondrial toxicity associated to ARV treatment in pregnancy [96]. In this framework, research on therapeutic intervention effective in reducing side effect risks of this life-saving regimen should be implemented. The present preclinical findings, besides providing a mechanistic hypothesis for the neurobehavioral effects of AZT, strongly suggest that preventive administration of LAC already shown to improve NRTI-induced polyneuropathy in HIV-1-positive patients [45]–[48] might also be effective in reducing the neurological side-effects of ARV therapy in fetus/newborn.

It is known that an excessive production of pro-inflammatory cytokines and free radicals might occur during HIV infection. Because pro-inflammatory cytokines and free radicals can travel through placenta to the fetus, they could contribute to increase the fetal oxidative stress induced by NRTI drugs. Nonetheless, our results highlight the importance of counteracting the neurotoxic effects of NRTIs in terms of rescue of neuronal function based on the maintenance of mitochondrial activity. Since LAC treatment has generally shown a good profile of safety and tolerability, our study strongly supports the rationale for the development of clinical trials aimed at investigating the efficacy of the administration of LAC to HIV-1 infected pregnant women treated with NRTIs to counteract potential adverse consequences on their children.
