# Correction: Transplacental Exposure to AZT Induces Adverse Neurochemical and Behavioral Effects in a Mouse Model: Protection by L-Acetylcarnitine

**DOI:** 10.1371/annotation/e675ab9e-a978-4f9d-a575-67a337964790

**Published:** 2013-10-04

**Authors:** Anna Rita Zuena, Chiara Giuli, Aldina Venerosi Pesciolini, Antonella Tramutola, Maria Antonietta Ajmone-Cat, Carlo Cinque, Giovanni Sebastiano Alemà, Angela Giovine, Gianfranco Peluso, Luisa Minghetti, Raffaella Nicolai, Gemma Calamandrei, Paola Casolini

There was an error in the affiliation for Maria Antonietta Ajmone-Cat. The correct affiliation is: Section of Experimental Neurology, Department of Cell Biology and Neurosciences, Istituto Superiore di Sanità, Rome, Italy 

